# Maternal diabetes impairs oxidative and inflammatory response in murine placenta

**DOI:** 10.1186/s40064-016-2180-y

**Published:** 2016-04-26

**Authors:** Mohamed I. Saad, Taha M. Abdelkhalek, Moustafa M. Saleh, Maha M. Haiba, Shady H. Tawfik, Maher A. Kamel

**Affiliations:** Department of Biochemistry, Medical Research Institute, 165 Elhorreya Avenue, P.O. Box 21561, Alexandria, Egypt; The Ritchie Centre, Hudson Institute of Medical Research, Monash University, Melbourne, VIC Australia; Department of Human Genetics, Medical Research Institute, Alexandria University, Alexandria, Egypt; Department of Molecular Medicine, University of Padova, Padua, Italy

**Keywords:** Diabetes, Placenta, Embryopathy, Birth defects, Oxidative stress

## Abstract

Placenta is the major exchange surface between mother and fetus and plays a pivotal role in fetal development. A better understanding of the mechanisms by which diabetes alters placental function may allow better management of diabetes pregnancies. In this study, we attempt to investigate the effect of diabetic milieu with and without malformation on placental function. In order to investigate the impact of diabetic pregnancy on oxidative stress, endothelial and vascular functions of placental tissue, we mated diabetic and non-diabetic female rats with normal male rats. At gestational day 17, we terminated pregnancy, assessed fetuses for malformations and isolated placenta for measurement of various parameters of placental function. Our results show that maternal diabetes induced a state of oxidative stress in placenta, which disrupts normal signaling, activating apoptosis, as well as perturbing endothelial and vascular placental functions. The coalescence of these insults on various levels of placental function could contribute to the pleiotropic nature of diabetes-induced placental stress.

## Background

Placenta is an evolutionary conserved organ present in almost all female mammal pregnancies (Wildman 2011). Placenta is essential for consummate pregnancy, and serves several roles including: secretion of hormones, interface to allow exchange of metabolites, oxygen and nutrients between maternal and fetal circulation, as well as modulating maternal immune system and fetal growth (John and Hemberger 2012). Placentogenesis is initiated early during mammalian pregnancy due to differentiation of trophoblasts from early embryonic stem cells. Nevertheless, complete layers of rats’ placenta are discerned from as early as gestational day (GD) 12 (De Rijk et al. 2002). Placental function increases during the late phase of pregnancy to compensate for the increase in fetal circulation (De Rijk et al. 2002). Therefore, GD 17 was chosen to assess placental function in its peak and to decrease confounding factors such as normal increase in placenta oxidative stress that occurs in later days of gestation.

Placenta secretes several growth factors including placenta growth factor (PIGF2), which promotes proliferation of placental vasculature and has an affinity to vascular endothelial growth factor (VEGF) receptors (Hauser and Weich 1993). PlGF-2 is a growth factor isoform that is secreted exclusively by umbilical cord endothelial cells and placenta (Hauser and Weich 1993). Soluble VEGF receptor1 (sVEGFR1) or soluble fms-like tyrosine kinase-1 (sFlt-1) is a splice variant of VEGFR1 and act as receptor for PIGF-2 expressed on endothelial cells and have several anti-angiogenic functions through several mechanisms (Wu et al. 2010).

Diabetes and/or hyperglycemia during pregnancy perturbs placental and fetal development (Castori 2013); however, the mechanism and contribution of defects introduced by diabetic milieu on placenta is not well established. Diabetes-induced embryopathy and malformations were established since early twentieth century (Castori 2013). The abnormal milieu instigated by diabetes and/or abnormal placental function may disturb the fetus on short term, but also may contribute to the development of disease later on in life (fetal origins of disease) (Calkins and Devaskar 2011). In order to better control and manage diabetic pregnancies to reduce embryopathies and possible adult effects, we need deeper understanding of the cellular and molecular mechanisms underlying diabetic sabotage of fetal development. Basic research using animal models has contributed a considerable amount of information about the manifestations of fetal abnormalities, but the effect of diabetes on placenta has not been thoroughly investigated.

Several cellular mechanisms contribute to the pathology of diabetic malformations including impaired endothelial function, increased apoptosis, and oxidative stress that disrupt organelle function as well as protein folding and processing (Gareskog et al. 2007). These apoptotic activities are mediated by increased expression and activity of cleaved caspase-3, which is regulated by high caspase-8 activity under hyperglycemic conditions (Yang et al. 2008). Moreover, hyperglycemia alters normal placental function by reducing expression of VEGF-A, VEGFR-1/Flt-1 and PIGF in placenta and serum of diabetic mothers. These perturbations might mediate the negative effects of diabetes on placental vasculature (Helske et al. 2001).

Hyperglycemia induces oxidative stress mainly through increased flux of NADH and FADH_2_ to electron transport chain that promote mitochondrial dysregulation and generation of superoxide radicals (Saad et al. 2015). Also, it impairs intracellular antioxidant defense by decreasing levels of reduced glutathione (GSH) and increasing oxidized glutathione (GSSG). Since glutathione system, the predominant intracellular antioxidant mechanism, is impaired and reactive oxygen species (ROS) production is increased; this imbalance induces a state of oxidative stress, which disrupts normal cellular signaling and leads to metabolic adverse effects of diabetic pregnancy (Sakamaki et al. 1999). Although ROS is involved in several physiological functions, the term of oxidative stress is reserved mainly for the pathological mechanism or effects (Sies 2015). One major direct effect of oxidative stress is oxidation of nitric oxide—a critical vasodilator and signaling molecule in early development—by reacting with ROS to form nitroperoxides, which in turn induce nitrosative stress. A proxy for nitrosative stress that may reflect levels of protein nitrosylation is nitrous and nitric oxides levels in tissues, collectively known as nitric oxide end products (NOx) (Jawerbaum et al. 2005). 8-Oxo-7,8-dihydro-2′-deoxyguanosine (8-oxo-dG) is a major biomarker of DNA damage induced by oxidative stress. It is formed by the reaction of OH radical with the DNA guanine base and usually result in point mutations (Cooke and Evans 2007a).

Cyclooxygenase enzymes COX-1 and COX-2 convert arachidonic acid into prostaglandins such as prostaglandin E_2_ (PGE_2_), while free radicals convert arachidonic acid into PGE_2_-like isoprostanes. In embryos of diabetic pregnancy, COX-2 activity and expression are reduced, which decrease PGE_2_ significantly and shift arachidonic acid metabolism into production of PGE_2_-like isoprostanes by ROS (El-Bassiouni et al. 2005). PGE_2_ protect embryos from the damages due to hyperglycemic conditions (Goto et al. 1992), while PGE2-like isoprostanes have damaging effects in animal models of diabetic pregnancy and embryos exposed to high glucose in culture (Wentzel and Eriksson 2002).

In this study, we investigate the impact of diabetic milieu on placental function of malformed and non-malformed fetuses, to study the effect of diabetes with and without malformations on placental function in late gestation. We assessed the impact of maternal diabetes through several parameters of vascular and oxidative processes in placenta. Our hypothesis is that diabetes affects placenta vascular, inflammatory and oxidative function during late gestation.

## Methods

### Experimental animals

The animal protocol was approved by the Institutional Animal Care and Use Committee at the Medical Research Institute—Alexandria University. Thirty female Local Wistar-derived strain rats were included in the experiment and had an initial average weight of 180 ± 20 g. Rats were given tap water and commercial pellet diet ad libitum. Animals were housed individually on a 12-h light/dark cycle and were kept at constant humidity and temperature (20 ± 5 °C).

### Induction of experimental diabetes

Diabetes was induced in adult female rats group (n = 15) through intraperitoneal injection of 55 mg/kg Streptozotocin (STZ; Sigma-Aldrich Chemical Co., Poole, UK) dissolved in sodium citrate buffer (0.01 M, pH 4.5) given daily over two successive days. An equivalent volume of buffer was administered to the control group (n = 15). Glucometer (Elite) was used for measuring glucose levels. Diabetes was confirmed by a blood glucose level >11 mmol/l measured 1 week after Streptozotocin injections.

### Mating

Female rats of both groups were mated with healthy non-diabetic males overnight. The presence of sperm mucus plug in the vagina the following morning signified pregnancy (GD 0).

### Sample collection and preparation

Female rats of both groups were culled by cervical dislocation on GD 17. In each sacrificed rat, the uterus was exposed by cesarean section and the number of implantations and resorptions were noted. The live embryos and their placentae were dissected out of the uterine horns, rinsed carefully in phosphate buffered saline (PBS). Overall growth and differentiation of the embryos were quantified by direct measurement of crown-to-rump length (CRL). The embryos were examined with regard to general morphology, and embryo and placentae were weighed. The occurrence of disturbed embryonic development was noted such as open neural tube, growth retardation and appearance of specific organs (head, ear, heart, legs, tail, and body rotation). Embryos were denoted as “malformed” if one or more of these structures exhibited an apparent anomaly. The weights of embryos and placentae were recorded. The placentae were snap-frozen in liquid nitrogen for subsequent analysis. Placenta was homogenized in 3 mL of 0.1 M Tris-HCl buffer, pH 7.4 containing 1 mM EDTA. Then the homogenates were centrifuged at 10,000*g* for 15 min at 4 °C. The supernatant was removed and stored at −80 °C for subsequent assays.

### Sample assays

COX enzymes activity assays were determined according to the procedure used by Kargman et al. (1996). Briefly, the activity of COX-1 was assessed in the standard and placental (control and diabetic) supernatants treated with COX-2 inhibitor, and compared with the activity of a mixture of both standard COX-1 and placental supernatant treated with COX-2 inhibitor. The kit (Cayman Chemical Company; Ann Arbor, MI, USA) includes isozyme-specific inhibitors for distinguishing COX-2 from COX-1 activity.

The concentration of PlGF-2 was determined quantitatively using EIA kit (R & D systems, Inc., Minneapolis, MN, USA) with a sensitivity of 8.38 pg/mL. The intra- and inter-assay coefficients of variation were 8 and 13 %, respectively. Placenta level of sVEGFR1/sFlt-1 was determined by competitive ELISA kit (Kamiya Biomedical Company, Seattle, WA, USA) according to manufacturer instructions. Caspase-3 activity was assayed by a method described by Thornberry (1994). Briefly, a fluorescently labeled substrate for caspase 3 was added and the rate of its degradation was assessed by ELISA. Oxidative stress parameters measured included thiobarbituric acid reactive substances (TBARS), which represents lipid peroxidation end-products and quantified as malondialdehyde (MDA). TBARS was assayed by a protocol developed by Draper and Hadley (1990). Briefly, this involves the reaction between thiobarbituric acid and MDA, which gives off a colored product assayed by colorimetry. While glutathione oxidation parameters were assayed by an enzymatic recycling assay developed by Griffith (1980). Shortly, GSH is oxidized by 5,5′-dithio-bis(2-nitrobenzoic acid) (DNTB) to from a product detected by colorimetry. Total glutathione level is detected by reducing all the GSSG resulting from the previous reaction using (NADPH and glutathione reductase) and repeating the initial assay again. Tissue content of vitamin C was assayed by colorimetry using Folin phenol reagent (Jagota and Dani 1982), vitamin E using HPLC (McCormick and Green 1996) and selenium by atomic absorption spectroscopy (AAS) (Herber and Stoeppler 1994) were also assayed in the placentae of all study groups. Protein content was determined using a modification of the method of Lowry et al. (1951). The specific activity of each enzyme was determined by dividing its activity by the protein concentration in the sample (U/mg protein).

Isolated DNA was washed twice with 70 % ethanol, dried, and dissolved in 200 μl of 10 mM Tris/HCl, 0.1 mM EDTA and 100 mM NaCl (pH 7.0). For enzymatic digestion, 100 units of DNase I were added per 200 μg DNA at 37 °C for 1 h. Then, 5 units of nuclease P1 and 30 μl of 10 mM ZnSO4 were added, and the mixture was incubated for 1 h. After re-adjusting the pH with 100 μl of 0.4 M Tris/HCl (pH 7.8) followed by the addition of three units alkaline phosphatase, the samples were incubated for 30 min. Enzymes were precipitated with acetone (5 vol), removed by centrifugation, and the supernatant was evaporated to dryness. The DNA hydrolysates were evaluated for 8-oxo-dG content using HT 8-oxo-dG ELISA Kit II (Trevigen, USA).

The nitrite and nitrate (NOx) concentration was determined by simple Griess reaction (Guevara et al. 1998). Briefly the assay consists of two steps: diazotization of sulphanilic acid with nitrite ion and coupling of this product with diamine, which results in a measurable pink metabolite that measured at 540 nm. Before diazotization reaction, all nitrate ions present in tissue were converted to nitrite ions by the action of NADPH-dependent nitrate reductase.

### Statistical analysis

All data are presented as mean ± SD. A one-way analysis of variance (ANOVA) with post hoc test (LSD) was performed on each normally distributed variable to compare the mean values. Differences were considered significant at P < 0.05. For pregnancy outcome, results were analyzed by Fisher’s exact test. All statistical analyses were performed using SPSS statistical software version 18 (SPSS, Chicago, IL, USA).

## Results

In this study, we investigated several parameters of oxidative stress, endothelial function, angiogenesis and apoptosis to get a better understanding of diabetes-induced pathological processes during late gestation.

Diabetes has profoundly negative effects on pregnancy outcome. Diabetic female rats had significantly higher number of resorptions and malformations but a lower number of viable fetuses, while normal females had no malformations and only two resorptions (Table 1). Various malformations were observed in fetuses of diabetic females; most commonly, growth retardation followed by mal-rotations and short mandible as well as head mal-development, absence of tail and open neural tube (Table 3). Moreover, crown rump length was significantly decreased in diabetic embryos regardless of their malformation status (Table 2). Placental weight was significantly higher in diabetic malformed and non-malformed fetuses relative to control pregnancies. On the contrary, embryo weight was significantly lower in diabetic malformed embryos compared to diabetic non-malformed and control embryos (Table 2).

**Table 1:** Pregnancy outcome of diabetic and control rats

Parameters	Control	Diabetic
No. of litters	9	13
Malformed litters	0	11*
% of malformed litters	0	85
Total no. of implantations	102	123
No. of implantations/litter	11	9
No. of viable embryos	100	98*
No. of resorption	2	25
% of resorption	2	20
No. of malformations	0	40

* Significantly different from control rats (P < 0.05) by Fisher’s exact test

**Table 2:** Fetal parameters and placental weight at gestational day 17

Parameters	Embryos from control mothers	Embryos from diabetic mothers	
		Non-malformed	Malformed
CRL (mm)	12.65 ± 0.21	10.89 ± 0.18*	9.76 ± 0.19*
Embryo weight (mg)	288.6 ± 4.21	222.3 ± 4.97*	209.5 ± 4.42*
Placental weight (mg)	187.8 ± 3.79	223.1 ± 3.23*	241.5 ± 3.66^#^

* Significantly different from embryos of control mothers (P < 0.05)

^#^Significantly different from non-malformed embryos of diabetic mothers (P < 0.05), by ANOVA

**Table 3:** The apparent malformation types in embryos of experimental diabetic pregnanacy

Malformation	Number of embryos	%
Growth retardation	21	52.5
Open neural tube	3	7.5
Maldevelopment of head	5	12.5
Absence of tail	4	10
Malrotation	13	32.5
Short mandible	6	15

**Fig. 1:**
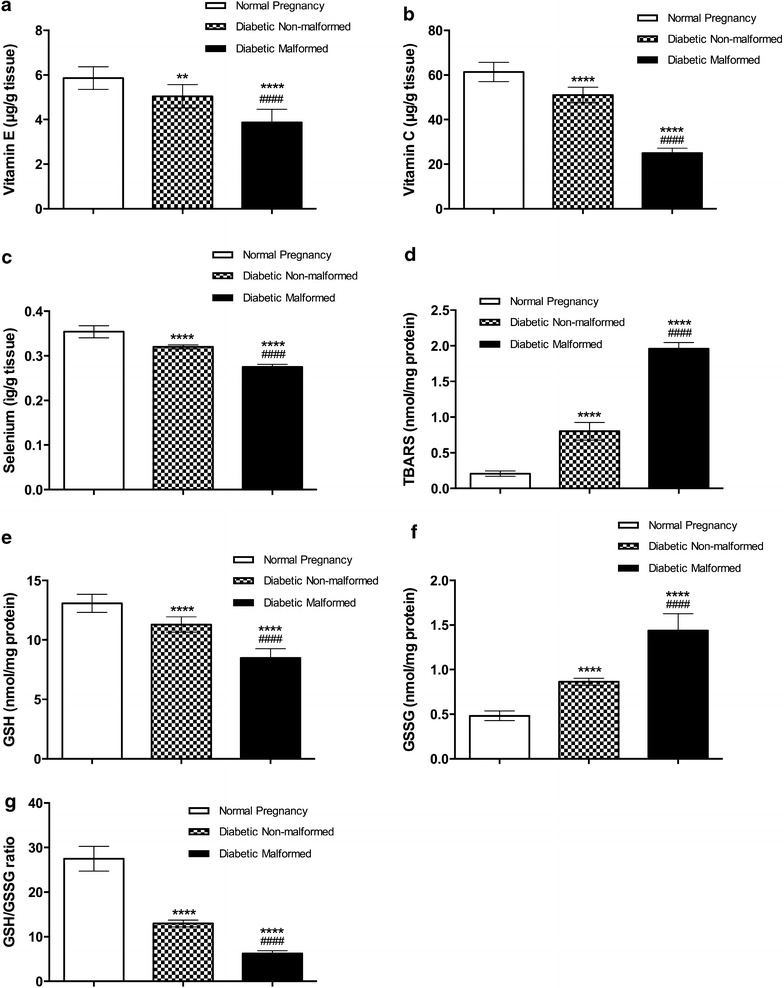
Impact of maternal diabetes on oxidative stress and antioxidants in placenta. Data are presented as mean ± SD (n = 15). *Statistically significant difference from controls, ^*#*^statistically significant difference from diabetic non-malformed, by ANOVA (P < 0.05)

At GD 17, non-malformed placentae from diabetic mothers had significantly lower levels of antioxidant vitamins (vitamins C and E), as well as antioxidant trace element, Selenium (cofactor of glutathione peroxidase and thioredoxin reductase) compared with controls. Placentae of malformed fetuses of diabetic mothers had even more significant decrease in the levels of vitamin C, vitamin E and Selenium compared with non-malformed diabetic and controls (Fig. 1). Markers of oxidative stress levels and ROS production were higher in placenta of diabetic mothers than controls. TBARS levels were significantly elevated in placentae of malformed diabetic compared to placentae of non-malformed embryos from diabetic mothers, and both were significantly elevated compared to controls (Fig. 1).

Levels of GSSG were significantly increased in placentae of malformed when compared with non-malformed diabetic fetuses, in which GSSG was higher than controls. The ratio of GSH/GSSG is a powerful indicator of the ROS production inside the cell and the ability of the cell to counteract oxidative stress. Both GSH and GSH/GSSG ratio were significantly diminished in placentae of malformed fetuses of diabetic mothers compared to both non-malformed and controls. Furthermore, non-malformed placentae of diabetic mothers had significantly reduced levels of GSH and GSH/GSSG ratio compared with controls (Fig. 1).

**Fig. 2:**
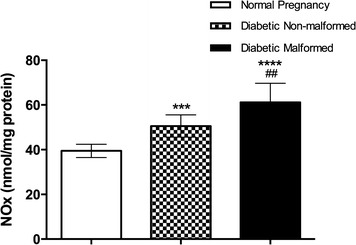
Levels of nitric oxide end products NOx (nitrates and nitrites) as markers of nitrosative stress in placenta. Data are presented as mean ± SD (n = 15). *Statistically significant difference from controls, ^#^statistically significant difference from diabetic non-malformed, by ANOVA (P < 0.05)

**Fig. 3:**
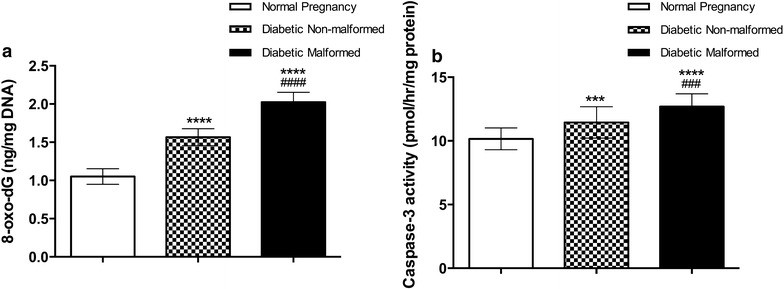
Levels of 8-oxo dG (DNA oxidation adduct) and cleaved caspase-3 activity as a measure of activated apoptosis in placenta. Data are presented as mean ± SD (n = 15). *Statistically significant difference from controls, ^#^statistically significant difference from diabetic non-malformed, by ANOVA (P < 0.05)

We found significantly elevated levels of NOx in placentae of malformed fetuses of diabetic mothers compared with non-malformed and controls. Moreover, NOx were increased in non-malformed diabetic placenta compared to controls (Fig. 2). Higher levels of NOx could indicate oxidative stress and endothelial dysfunction. 8-oxo-dG levels were significantly higher in placentae of malformed and non-malformed fetuses (Fig. 3).

**Fig. 4:**
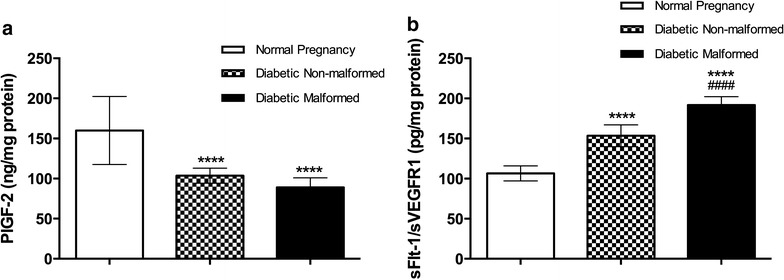
PIGF-2 and sFlt-1/sVEGFR1 expression in placenta indicate vascular and endothelial functions. Data are presented as mean ± SD (n = 15). *Statistically significant difference from controls, ^#^statistically significant difference from diabetic non-malformed, by ANOVA (P < 0.05)

Placental growth factor 2 (PlGF-2) levels were significantly reduced in placentae of fetuses of diabetic mothers compared with controls regardless of malformation status. Placental levels of sFlt-1 were significantly higher in diabetic fetuses placenta regardless of malformation status (Fig. 4).

**Fig. 5:**
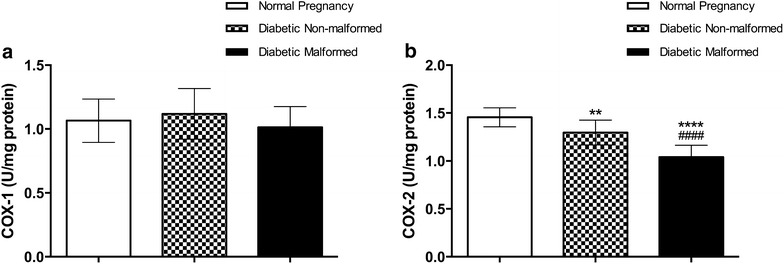
Activity of cyclooxygenases (COX-1 and COX-2) in placenta to assess inflammatory response and prostaglandin production in placenta. Data are presented as mean ± SD (n = 15). *Statistically significant difference from controls, ^#^statistically significant difference from diabetic non-malformed, by ANOVA (P < 0.05)

Caspase-3 is a critical death protease, indispensable for apoptotic chromatin condensation and DNA fragmentation. Caspase-3 activity is significantly increased in placentae of malformed compared with that of non-malformed diabetic offspring, which have higher caspase-3 activity compared with controls (Fig. 3). The cyclooxygenase isoenzymes, COX-1 and COX-2, catalyze the oxidative cyclization of the central 5 carbons within 20 carbon polyunsaturated fatty acid (arachidonic acid) to form prostaglandins, thromboxane, and levuloglandins. COX-1 is produced constitutively while COX-2 is inducible (e.g. by inflammation). COX-1 enzyme activity did not differ significantly between malformed, non-malformed or control fetuses’ placentae. However, inducible COX-2 enzyme activity was significantly diminished in malformed compared to non-malformed fetuses of diabetic mothers and controls. COX-2 activity was also significantly reduced in non-malformed fetuses’ placenta when compared with controls (Fig. 5).

## Discussion

Diabetes mellitus during pregnancy affects the developmental milieu, jeopardizes the normal fetal development and increases the risk of various adult onset morbidities (Calkins and Devaskar 2011). We have previously demonstrated that diabetes during pregnancy increases oxidative stress in various fetal tissues including placenta (Kamel et al. 2014). However, the effect of maternal diabetes on placental inflammation, vascular and oxidative stress markers specifically, was not studied thoroughly. In this study we demonstrate that oxidative stress is significantly elevated in placenta of diabetic pregnancies regardless of malformation status of the fetus.

Our results show clearly that diabetic pregnancy placentae exhibit increased oxidative stress. Moreover, the intra-cellular antioxidant defense mechanisms, as characterized by Glutathione oxidation parameters, were diminished in placentae from diabetic pregnancies. Normal placentae show a significant increase in oxidative stress that is instrumental to their normal development (Schafer and Buettner 2001). However, as pregnancy proceeds, the level of oxidative stress decrease and anti-oxidant levels increase (Basu et al. 2015). This suggests that diabetes may disturb normal milieu of placenta through increasing oxidative stress.

Other anti-oxidant systems were also diminished in placentae of diabetic pregnancies regardless of their malformation status. Moreover, anti-oxidant vitamins levels in tissues further corroborate the findings indicating increased oxidative stress in placentae of diabetic pregnancies. Whether the observed increase in oxidative stress is caused by the diminished anti-oxidants levels in placenta or vice versa cannot be elucidated form our work. However, it must be noted that normal pregnancies exhibit reduced serum levels of vitamin E compared to non-pregnant mothers, as pregnancy is a physiological condition associated with increased oxidative burden (Yoshioka et al. 1987). Moreover, the level of vitamin E in fetal tissues is related to that in corresponding maternal tissues and is amenable to the type of diet ingested by the mother (Yoshioka et al. 1987).

The increase in oxidative stress might have caused several deleterious effects on various cellular targets. TBARS levels were significantly elevated in a similar pattern as glutathione compounds, which illustrate the increased peroxidation of lipids in the affected placentae. Moreover, markers of DNA oxidation in diabetic placentae were also significantly increased indicating that the increased oxidative stress was high enough to cause such damage. DNA oxidation may hinder normal function of cells, which may be explained by the effect of these modifications in increasing mutation rate of affected cells, specifically transversions (Cooke and Evans 2007b).

Furthermore, nitric oxide levels show a similar pattern of elevation as that observed in 8-oxo-dG, which may reflect increased protein nitrosylation in placenta. Nitrative stress or increased protein nitrosylation is a hallmark of complicated pregnancies such as those with pre-eclampsia or gestational diabetes, and the nitrosylation of DNA and proteins hamper the normal function of placenta (Lyall et al. 1998; Myatt 2010). Furthermore, the increased damage inflicted by the increased oxidative stress was also evident by the increased activity of the apoptosis marker Caspase-3 observed in our results. Our results hint to the possibility that disturbed placental milieu may induce fetal programming of adult disease. At late gestation, several important physiological changes occur that may be affected by the disturbed milieu.

In addition to the parameters of oxidative stress and anti-oxidant measured, we also measured the activity of various enzymes and growth factors in placenta. Our results are in concordance with previous studies that demonstrated association of sFlt-1 elevation with diabetes or pre-eclampsia (Salim et al. 2009; Wu et al. 2010). Given the known functions of PIGF-2 and sFlt-1, we can hypothesize that the observed pattern reflect an abnormality in the fetal or placental vasculature. However, to confirm such a hypothesis, thorough analysis of fetal vasculature must be conducted.

The downstream effects of the observed increase in placental oxidative stress were also observed. The content of cyclooxygenase isozymes was altered in placentae of diabetic malformed and non-malformed fetuses. Our results are in concordance with previous work published (Al-Matubsi et al. 2010) were COX-2 was significantly reduced in malformed diabetic compared to non-malformed diabetic placentae; however, these measurements were conducted on placentae extracted in earlier day of gestation. A major discrepancy between both results is that in our measurements COX-1 and COX-2 levels were comparable, while in Al-Matubsi and colleagues work, COX-1 levels were relatively small compared to COX-2. In absence of diabetes, oxidative stress induces expression of COX-2 in murine placenta (Burdon et al. 2007). However, in our results despite increased oxidative stress, COX-2 expression was significantly reduced in placenta, which may suggest that diabetes might have disrupted normal response to oxidative stress.

Our results are in harmony with previous observations conducted during different times of gestation. Diabetes in pregnancy regardless of malformation status is associated with increased oxidative stress and disturbed placental function. The exact mechanism by which oxidative stress disrupts normal placental function is not known but may be a result of a complex menagerie of cellular insults such as DNA mutation, protein oxidation and nitrosylation as well as apoptosis. Moreover, other conditions related to diabetes such as obesity, may contribute to the observed placental dysfunction. Although absence of fetal malformation does not rule out placental alteration, we did not observe any placental alteration in diabetic embryos (malformed and non-malformed). However, to confirm or delineate the contribution of these factors, further studies should be conducted.

## Conclusion

Maternal diabetes is associated with a state of oxidative stress, sparseness of various antioxidants and growth factors that are critical for endothelial and vascular functions, and increased apoptotic activity in placenta. Whether theses perturbations are causally related or just coincidental with the effects of high glucose on placental function is still unclear. Further studies are needed to confirm and illustrate the molecular mechanisms of these abnormalities.

## Abbreviations

T2DM: type 2 diabetes mellitus
GSH: glutathione
ROS: reactive oxygen species
RNS: reactive nitrogen species
8-oxo-dG: 8-oxo-7,8-dihydro-2′-deoxyguanosine
TBARS: thiobarbituric acid reactive substances
MDA: malondialdehyde
CRL: crown-rump length
VEGF-A: vascular endothelial growth factor-A
VEGFR-1/Flt-1: vascular endothelial growth factor receptor
PIGF: placenta growth factor
NOx: nitric oxide end products
COX-1: cyclooxygenase isoenzyme-1
COX-2: cyclooxygenase isoenzyme-2
PGE_2_: prostaglandin E_2_
NADH_2_: nicotinamide adenine dinucleotide
FADH_2_: flavin adenine dinucleotide
